# Topics of the Month

**Published:** 1894-03

**Authors:** 


### TOPICS OF THE MONTH.

Dr. Nicholas Senn, of Chicago, has presented his collection of medical books, valued at about $50,000, to the Newberry Public Library. This is in line with similar benefactions that we have lately mentioned in these columns, and is to be commended as one of the best methods of increasing the usefulness of medical literature. Private libraries rarely benefit anybody excepting their owners, whereas a public library is accessible to the masses, and thus benefits mankind. Professor Senn has been engaged a number of years in gathering his library, many of its most valuable books having been purchased from the executors of Dr. William Baum, Professor of Surgery in the University of Göttingen. These were valued at over $40,000, and Dr. Senn was fortunate in securing this collection, which contained a number of antiquarian volumes separately valued at enormous prices. Dr. Senn deserves the thanks of the medical profession everywhere for this magnificent gift.

---

Medical stenography is becoming an important specialty among shorthand writers. There is a constantly growing necessity among physicians for the services of such as are competent to receive dictation in medical terms not only for purposes of letter-writing, but also for the writing of medical papers that are to be read before societies or published in magazines. Then, again, authors of text-books or treatises find it an absolute necessity to employ a competent medical shorthand writer in order to keep pace with the demands of the publishers for copy.

Miss Mary L. Danforth, of Buffalo, is a competent medical stenographer, who offers her services to physicians in this city. Her card appears on page xxiii. in our advertising columns, to which we invite attention.

---

The smart medical student is again on hand with his little bill before the Legislature relating to the Medical Examiners' law.

This time he simply asks that the fees be reduced from $25 to $5. This looks very innocent at first sight, but when it is understood that during the first two academic years of the operations of the law governing the State Medical Examining and Licensing Boards, the expenses were such as to leave a balance to each examiner of only $123 a year, it can be easily seen that such an amendment as is proposed by the students would render the law inoperative, for it would reduce the receipts far below the expenses.

It would seem to us more logical for the students to petition the faculties of the medical colleges to abrogate the diploma fees that are universally charged. The diploma is merely a certificate of study, and might be readily furnished by the colleges for a fee of five dollars that would more than cover its cost, not to say its value; whereas, the State license once obtained is a diploma that has legal recognition in all the states as well as in many foreign countries. The students evidently do not comprehend the value of the license that they are struggling to nullify, else they would change front and direct their united forces towards the reduction of the college diploma fees to which we have referred.

---

THE COMMITTEE on legislation of the Medical Society of the State of New York, consisting of Drs. D. B. St. John Roosa, Daniel Lewis and D. V. O'Leary, has entered protest before the Senate Committee on Public Health against the passage of a bill in which the medical students are asking for a reduction of the State fees for examination. Now that so many States are requiring separate examinations, the latest of all being Pennsylvania, it is amazing to us that an intelligent legislature should listen one moment to appeals, or waste an instant of valuable time in the consideration of bills, that look to a nullification of the excellent medical practice act now in force.

Shall New York drop behind her sister commonwealths in the reform movements that are taking possession of all states of the Union with reference to medical education? It seems to us that these annual attempts of the students to resist, in one form or another, the application of this law, is a confession on their part of ignorance, timidity or cowardice. If they are well equipped, why should they hesitate to take the examination? If not well equipped, why have they been allowed to obtain the faculty diploma?

If the State should be persuaded into the necessity of relieving the medical student of a portion of his fees, let it either render it unlawful for the college faculties to charge a diploma fee in excess of $5 or else pay to the Regents of the University the State fees for examinations.

---

THE SUBJECT of male nurses has recently been commented upon by Dr. Charles H. Stowell in *Food.* We have for a long time been of the opinion that the male nurse had very small place in the care of the sick. This is certainly true outside of the insane hospitals or prisons, or in a few exceptional cases where mere muscular power is needed. In private homes, certainly, the male nurse is rarely required. In these days of trained nurses there is no comparison between the male and the female nurse as regards usefulness in the sick room.

There are many diseases, for example, typhoid fever, where nursing has a larger place than medicine, and today no reputable physician would conduct such a case without obtaining the services of a trustworthy trained nurse to carry out his instructions and to do the thousand and one things so needful to establish convalescence, that only her thoughtful brain and delicate hand are capable of accomplishing. We believe there has been no stronger evidence of progress in medical science in the last fifteen or twenty years than in the training of nurses and in their extensive employment in the sick-room.

---

THE ANNUAL report of the resident physician of Brigham Hall, a hospital for the insane that was established in 1855 at Canandaigua, has come to our table. It is a neat brochure with numerous illustrations from photographs, and bespeaks the progress that is making in private institutions of this character. Dr. D. R. Burrell, the physician in charge, has had a large experience in the treatment of the insane, and the hospital over which he presides is justly one of the most celebrated private institutions for the care of this class of patients in this country. Its location, too, is such as to commend it from a general sanitary point of view.

---

SURGEON-GENERAL GEORGE M. STERNBERG, United States Army, announces the convening of an Examining Board in Washington, April 15, 1894, for the purpose of passing upon the qualifications of candidates seeking appointment in the medical corps of the United States army. The examination is very rigid, and, as a rule, not more than one out of five to ten succeeds at the examination. The pay, too, is entirely inadequate to command the skill that a well-educated practitioner should possess. It is probable, however, that the improved standards of medical education adopted by the colleges, consequent upon and resultant from the establishment of separate State medical examining and licensing boards, will serve to equip candidates better, so that rejections will now be fewer by the army and navy examining boards. We would not discourage young men from attempting these examinations, but rather would encourage them to adequately prepare for them.

---

The State of Pennsylvania has finally fallen into line with many other States in the Union, regarding separate State medical examination for license. Under the Act of May 18, 1893, the Governor has appointed the following boards of State Medical Examiners:

*Representing the Regular Profession*—H. G. McCormick, Williamsport, three years; Henry Beates, Jr., Philadelphia, three years; W. J. R. Kline, Greensburg, three years; A. H. Hulshizer, Philadelphia, two years; N. S. Foster, Pittsburg, two years; J. E. Silliman, Erie, one year; Samuel W. Latta, Philadelphia, one year.

*Homeopathic*—C. S. Middleton, Philadelphia, three years; Hugh Piteairn, Harrisburg, three years; Isaac G. Smedley, Philadelphia, three years; Edward Cranch, Erie, two years; C. F. Bingamen, Pittsburg, two years; Augustus Korndoerfer, Philadelphia, one year; J. F. Cooper, Allegheny, one year.

*Eclectic*—H. Yeagley, Lancaster, three years; Augustus Niles, Wellsboro, three years; L. B. O'Neale, Mechanicsburg, three years; H. B. Piper, Tyrone, two years; J. R. Borland, Franklin, two years; W. H. Blake, Philadelphia, one year; A. B. Woodward, Tunkhannock, one year.

The Act also established a Medical Council, consisting of the Lieutenant-Governor, Attorney-General, Secretary of Internal Affairs, Superintendent of Public Instruction, President of the State Board of Health, and presidents of the three Boards of examiners appointed. The members of this council receive no salary, except the secretary and treasurer, who will receive not over $500. This council will meet twice a year, and supervise the examinations of the State boards and issue licenses to practice medicine and surgery. The expenses of the boards of examiners, it is provided, shall be paid from fees, and, if any surplus above expenses shall remain at the end of any year, it shall be apportioned among the examiners *pro rata*, according to the number of candidates examined by each. The first meeting of the examining boards will be held on the first Tuesday of April.

---

Medical politics in medical societies seems to disturb the *American Lancet* in the extreme. Especially is this the case with reference to the Medical Society of the State of New York, but it is not at all disturbed by the fact that the American Medical Association is a hotbed of medical politics, not to mention some politics that are not medical. Those who are most familiar with the workings of the Medical Society of the State of New York are aware that it was never freer from politics than during the last ten years. Again, these same persons are aware that the society never did any better scientific work, whether judged by quality or quantity, than during this same period. It would seem to us that it is ill-timed for a person outside the State, who does not even attend the meetings of the New York Society, to speak in disparagement of the good work that it accomplishes. Notwithstanding this criticism of the *Lancet*, we asseverate here and now, that the Medical Society of the State of New York is flourishing to a degree hitherto unknown, financially, scientifically and numerically, and it is willing to have its work compared with that of any other state medical organization, not doubting that the verdict will be favorable to its own record.

---

The action of the State Lunacy Commission has been a subject of criticism for some time past, not only in medical, but in lay journals. The resolutions introduced by Dr. Eugene Beach, of Gloversville, at the late session of the Medical Society of the State of New York, condemnatory of the centralized management of the State hospitals for the insane, was a just expression of an indignant profession regarding the conduct of the lunacy commission in general, and of the exercise of its political powers in particular. We trust the legislative committee of the society will be able to solve the problem through amendments to the existing law that will be satisfactory to all interests concerned.

THE FINANCES of the free kindergartens in Buffalo have become so strained in the carrying on of the present work, that in order to extend it proportionately to the increase in the population of the city, an appeal is made to public-spirited citizens, professional men and all who are interested in the progress of this important method of educating the young. A proposition has been made by Mr. John G. Milburn to secure the coöperation of the lawyers of Buffalo to raise a sum sufficient to maintain one kindergarten, provided the physicians will unite to sustain another. This means the pledging of from $1,000 to $1,200 a year, and it is much desired on the part of the kindergarten managers that this pledge be made for three years. If it should prove inexpedient for physicians to pledge $1,000, they possibly may be able to raise $600, which will support a kindergarten exclusive of the salaries which would be paid by the city. To aid this charity, the JOURNAL will receive any subscriptions, or they may be sent to Dr. James Wright Putnam, 388 Franklin street.

---

DR. GEORGE H. ROHÉ, Superintendent of the Maryland Hospital for the Insane, publishes in his annual report for 1893, a continuation of his observations on the relation of pelvic disease and psychical disturbances in women and the results of such observations. From advance sheets, kindly furnished the JOURNAL, we observe a table of twenty-two operations made by Dr. Rohé on the pelvic organs of insane women, and a review of the table shows that even in apparently the most hopeless case a beneficial effect upon the mental functions is obtained by the removal of a persistent source of local irritation. In one case of hystero-epilepsy with violent maniacal attacks lasting during a period of eight years, complete recovery was obtained. In this case both ovaries were cystic, and to the removal of the uterine appendages may be credited the cure. Dr. Rohé has been subjected to criticism for his work in the direction indicated, but his critics generally know little of the great advances made by modern gynecology, and are even more ignorant of the results of recent studies of mental pathology. The facts presented in this report will be regarded by all unprejudiced minds as a justification of the course pursued by Dr. Rohé in the conduct of these trying cases.

---

THE ANNUAL report of the Rhode Island Hospital for 1893 possesses a point of great interest, to which we desire to call attention, in the hope that it may stimulate some public-spirited citizen of Buffalo to imitate the example of Mr. George I. Chace, deceased, who was for many years president of the institution. Upon his death, Mr. Chace made certain charitable bequests to be paid upon the decease of his wife, including one of $12,000 for the establishment of free beds in the Rhode Island Hospital. He also directed that one-half of the remainder of his estate should go to such objects of charity as his wife might name by will, expressing the hope that the hospital would share in her remembrance. Mrs. Chace died December 25, 1892, and directed that, after the payment of certain sums, one-half of the remainder should go to the hospital for the erection of a building for the accommodation of the training school for nurses. The whole of this generous bequest has been devoted to the construction of a new building bearing the name of George Ide Chace, and used as a nurses' home. It is a handsome three-story structure with balconies in front, and is every way adapted to the purpose named. In these days of training schools and trained nurses, it is essential that these useful, not to say indispensable, young women should be provided with suitable homes adjacent to, but separate from, the hospital training schools. Let somebody in Buffalo, reading of the example set by Mr. George Ide Chace, go and do likewise.

